# Telemedicine Is Becoming an Increasingly Popular Way to Resolve the Unequal Distribution of Healthcare Resources: Evidence From China

**DOI:** 10.3389/fpubh.2022.916303

**Published:** 2022-07-06

**Authors:** Jinghong Gao, Chaolin Fan, Baozhan Chen, Zhaohan Fan, Lifeng Li, Linlin Wang, Qianqian Ma, Xianying He, Yunkai Zhai, Jie Zhao

**Affiliations:** ^1^The First Affiliated Hospital of Zhengzhou University, Zhengzhou, China; ^2^National Engineering Laboratory for Internet Medical Systems and Applications, Zhengzhou, China; ^3^Henan Province Telemedicine Center of China, National Telemedicine Center of China, Zhengzhou, China; ^4^Management Engineering School, Zhengzhou University, Zhengzhou, China

**Keywords:** telemedicine, necessity, development history, scale, operation procedure

## Abstract

**Background:**

Few studies focused on the general situation of telemedicine in China.

**Objectives:**

The purpose of this review is to investigate telemedicine in China, from the aspects of necessity, history, scale, and operation procedure, to improve the further development and implementation of telemedicine service.

**Methods:**

A literature search for peer-reviewed studies was conducted using the primary electronic databases. Additional documents from the official websites of Chinese government departments involved telemedicine was also collected. We extracted telemedicine related information focused on China from the final retrieved materials, and the general situation of telemedicine was drawn.

**Results:**

In China, telemedicine offers a feasible solution to the unequal allocation of healthcare resources, which makes telemedicine increasingly become an important alternative to close the gap between rural and urban in the capability and quality of medical services. China initiated telemedicine in the late 1980s. In 2018, China's telemedicine network has covered more than 3,000 hospitals across the country. As of 2019, almost all of the 31 provinces and municipalities in mainland have established regional telemedicine centers, and the market size of telemedicine reached about USD 2.68 billion. Based on the telemedicine network, remote rural patients can apply for healthcare services of top-tier urban hospitals through local county-level medical institutions.

**Conclusions:**

Through improving the capacity, quality, and efficiency of healthcare in underserved areas, and reducing the unequal distribution of medical resources, telemedicine can help solve the problems of the difficulty and high cost to access to medical services in China.

## Introduction

A major challenge faced by both developed and developing countries today is the inequity of access to healthcare resources. Based on information and communication technologies (ICTs), telemedicine offers a potential solution to the problem by providing cross-regional, accessible, and high-quality healthcare services (1, 2). Telemedicine can overcome geographical and temporal barriers, and help healthcare professionals exchange valid information and deliver medical services (1, 3). Telemedicine, a term firstly coined in the 1970s, which generally refers to the use of modern ICTs to increase the access of medical institutions, physicians, and patients to healthcare resources and medical information for the prevention, diagnosis, and treatment of diseases, response of major public health emergency, continuous education of medical staff, interdisciplinary research, administration and effectiveness assessment, and others in the interests of improving public health (1, 4, 5).

In general, there are two basic forms of telemedicine: synchronous and asynchronous, which are classified based on the timing of information transmission and the relationships between the individuals involved (1, 3). The former requires that the involved clinicians and patients are connected at the same time and exchange information simultaneously, such as live two-way interactive videoconferencing. In contrast, the latter involves the asynchronous communication and exchange of pre-recorded medical information, such as clinical examination reports, images, and video recordings, between different individuals separated by distance and time, as in the case of email or text message (1, 6). This latter approach is known as “store-and-forward.” For both synchronous and asynchronous telemedicine, necessary information can be transmitted in a variety of media, such as figure, audio, video, still picture, and text. In practice, these two telemedicine forms may be employed one or both with or without intermittent in-person consultations based on clinical and individual needs, which have been applied to various services in diverse settings, including teleconsultation, teleradiology, telemonitoring, teledermatology, and telepathology (1–3, 7). For instance, as one of the typical telemedicine service that use of ICTs to transmit medical information (e.g., photos or videos) concerning skin conditions for the purpose of teleconsultation and interpretation, teledermatology may be classified into two main modalities (1). In the modality store-and-forward, the local referring doctor firstly sends materials and description of a medical case to an expert of top-tier hospital, who sends back an interpretation and opinion regarding diagnosis advice and optimal treatment after a variable time interval. For the synchronous modality, individuals involved can exchange information and conduct dermatological teleconsultation through real-time two-way interactive videoconferencing.

As an open, sharing, and continuously evolving science, telemedicine constantly incorporates new advancements in ICTs and adapts to the contexts of socioeconomic development and changing public health needs to achieve the key purpose that the cross-regional and without time limited delivery of medical services and exchange of information (1, 8). Compare to traditional medical patterns, telemedicine with some typical elements should be highlighted, including (1) various types of ICTs are employed; (2) main purpose is to provide clinical support; (3) can overcome area and temporal limitations, and allow physicians to reach patients in different physical locations; (4) with the potential to enhance access to healthcare and improve patients' outcomes; (5) may alleviate the shortage of healthcare professionals and medical resources; (6) is usually, although not always, brings cost saving for both patients and healthcare facilities (1, 2, 7, 9). With these characteristics, to date, telemedicine has been used to a wide array of services in radiology, dermatology, cardiology, endocrinology, obstetrics, nephrology, neurology, gastroenterology, psychiatry, cardiovasology, and ophthalmology (8, 10–15). In recent years, the simultaneous advance and maturation of multiple ICTs has provided an unprecedented opportunity for further development and implementation of telemedicine. These technology innovations, including artificial intelligence (AI), wearable and implantable sensing technologies, 5th generation mobile networks (5G), Internet of Things, cloud computing and platform, and block-chains, are creating an inter-dependent ecosystem for new opportunities in telemedicine (2, 7, 16–18).

It is increasingly realized that telemedicine generally has the potential to increase access to healthcare services, make the most of scarce medical resources, improve clinical diagnosis, treatment and care of diseases, and advance the health of individuals. This is particularly true for the regions that traditionally suffer from lack of access to healthcare, such as remote villages, mountainous areas, isolated islands, or underserved communities, especially in developing countries (1, 19). In the United States, a non-profit American Telemedicine Association (ATA) was founded in 1993, which is committed to ensuring that everyone has opportunities and access to safe, affordable, and appropriate healthcare services when and where they need it. To date, the ATA has included more than 400 partner organizations and alliances, and there are more than 60% of health service organizations and 50% of hospitals have integrated telemedicine into routine medical services (20). In Europe, telemedicine has also been becoming one of the priority areas on the political agenda. It has been reported that more than 50 countries in Europe have established telemedicine systems, and the application fields include cardiology, radiology, ophthalmology, stomatology, emergency, monitoring, and health management (4, 21). In Brazil, telemedicine has been emerged since the early 1990s in a decentralized and fragmented manner in health, teaching, and research (22, 23). Due to the expansive geographies, with thousands of isolated, difficult-to-access locations, unequal distribution of medical resources, and uneven levels of health professionals, Brazil becomes a country of unique opportunities for telemedicine development and application (24–26). In order to minimize regional inequalities in the distribution of medical resources and specialists, reduce unnecessary referrals, establish continuing education for medical staff and, thus, obtain better cost-effectiveness and services quality, in 2007, the Ministry of Health formed the Telehealth Brazil Program, which was renamed National Telehealth Program Brazil Networks in 2011 (23, 27). Currently, for the program there are 26 Telehealth centers in 23 states serving 3,417 cities, involving teleconsultation, telediagnosis, telemonitoring, and tele-education (24, 27, 28). However, in Brazil, direct communication with patients such as teleconsultation remains forbidden. This situation only recently changed due to the outbreak of coronavirus disease 2019 (COVID-19). By the middle of April 2020, the Ministry of Health published a specific ordinance to authorize the use of telemedicine, during the COVID-19 pandemic, in any healthcare activities in Brazil (23, 29). Given the continuous pandemic of COVID-19, similar situations of quantum leap development and applications of telemedicine were also observed in many other countries around the world (30–32).

In China, telemedicine is a relatively new approach to healthcare delivery that is under rapidly developing. Various telemedicine networks covered different level hospitals have been launched to provide telemedicine services, such as teleconsultations, remote specialty diagnoses (e.g., tele-diagnosis of imaging results, tele-pathology, and tele-electrocardiograms), and remote education of medical staff (5, 33, 34). Telemedicine in China has been improving the dissemination of high-quality healthcare resources from the cities to remote rural areas, providing equivalent healthcare services in cities and underserved regions, and promoting China's hierarchical medical systems (34). However, to date there has no study tried to investigate the situation of telemedicine comprehensively in China, especially from the perspectives of both national and regional levels. Thus, in the current scoping review, we first investigate the necessity, development history, and scale of telemedicine in China at a national level. Second, we summarize the general operation procedure of telemedicine services in healthcare facilities in China, focusing on the information from provincial telemedicine centers. Lastly, taking the Henan Province Telemedicine Center of China (HTCC) as an example, from regional level we examine the specific telemedicine services and the corresponding effectiveness. To the best of our knowledge, this is the first comprehensive account of telemedicine in China, at both national and regional levels, and we hope the findings will help improve the further development and implementation of telemedicine service in China and provide significant references for other regions worldwide.

## Methods

### Study Design

This scoping review was conducted consistent with the guidance of the PRISMA-ScR (Preferred Reporting Items for Systematic Reviews and Meta-Analyses Extension for Scoping Reviews) checklist (35, 36). A scoping review was selected to identify and summarize the existing evidence about the development and implementation of telemedicine in China because it allows a general and comprehensive approach to the subject.

### Search Strategy

A literature search for peer-reviewed studies published up to 2020 was conducted using the electronic databases PubMed, China National Knowledge Infrastructure (CNKI), and Wanfang Data. Relevant reports from the World Health Organization (WHO) were also searched for further information. Our search used the following Medical Subject Headings (MeSH terms) from the US National Library of Medicine and key words: “telemedicine,” “telehealth,” “teleconsultation,” “health unfairness,” “healthcare distribution,” “remote specialty diagnoses,” “remote education,” “China,” and “developing country.” In order to facilitate an effective search, the search terms were adapted to the different databases. In addition, a comprehensive search from the official websites, government reports, public documents, yearbooks, and announcements of the National Health Commission of China (NHCC), National Bureau of Statistics, Henan Province Health Commission (HPHC), and HTCC was performed on 15 April 2020. References in the retrieved materials were examined and necessary manual searches were further performed.

### Eligibility Criteria

Both English and Chinese language articles were included in the initial search. The PICO criteria described in PRISMA was followed in this study. The identified materials were screened and selected in accordance with the pre-specified inclusion criteria, including (1) the development and implementation of telemedicine was involving the public in China; (2) telemedicine services such as teleconsultation, remote specialty diagnoses, or distance education were the studied factor(s); (3) the characteristics, advantages, or effectiveness of telemedicine compare to traditional medical services were investigated; and (4) the necessity, history, scale, or operation procedure of telemedicine were discussed.

### Data Extraction and Synthesis

For the retrieved materials, the telemedicine related information focused on China was extracted using a pre-designed data extraction form. Two authors (JHG and CLF) extracted data independently from the final selected references and the following key information was obtained: issues of medical services, the distribution of healthcare resources, necessity, development history, and scale of telemedicine, procedure of telemedicine service, challenges faced by telemedicine, and future work and directions of telemedicine. The information was then cross-checked and discrepancies were discussed by the data extractors and resolved through consensus among all authors. A comprehensive summary of the situation of telemedicine in China was finally drawn, at both the national and provincial levels.

## Results

**Figure 1:**
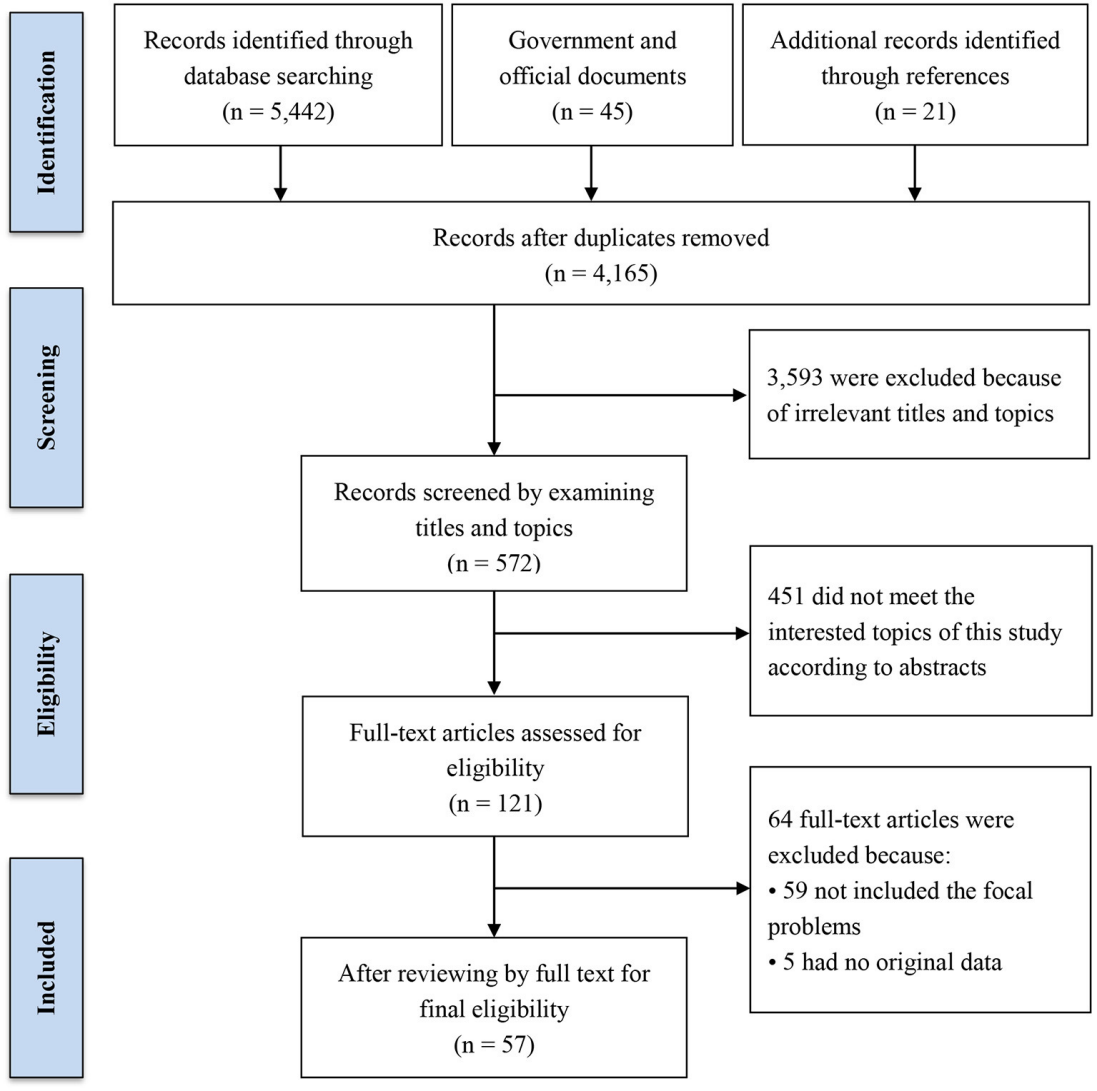
Flow chart of screening process of the literature search.

A total of 5,442 peer-reviewed records were identified from the databases, 45 official and government reports and documents were obtained, and 21 additional articles or documents were retrieved through references. After removing duplicates, the titles and abstracts of the preliminary identified materials were scanned for relevance. Based on the PICO criteria and topics of this review, materials that did not involve the aspects of necessity, history, scale, operation procedure, challenges, and future directions of telemedicine in China were excluded. Then, 121 materials were screened for a full-text examination and 57 articles or documents were retained (Figure 1).

### The Necessity of Telemedicine

**Figure 2:**
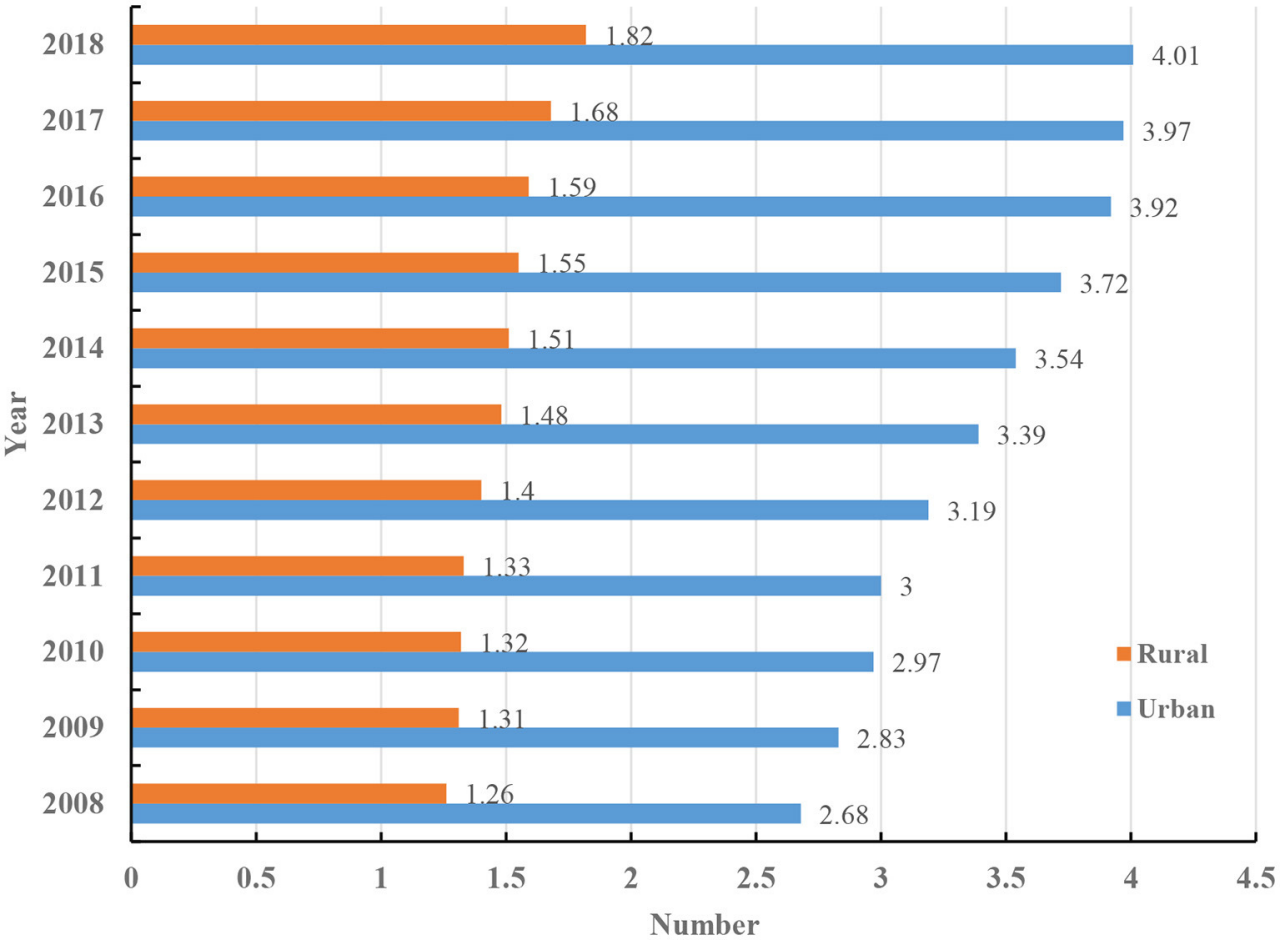
The number of practicing (assistant) physicians per thousand in urban and rural regions in China.

China's development gap between urban and rural regions is huge. For example, although 42.65% of China's population lives in rural areas, about 80% of China's medical resources are concentrated in the cities, two-thirds of which are in megacities (37). This geographically uneven distribution of medical resources has created relatively poor healthcare in remote rural areas. In 2018, compared with the figure 4.01 in the urban, the number of practicing (assistant) physicians per thousand in rural regions in China was only 1.82 (Figure 2) (38). Rural patients with intractable diseases often need to go to distant top-level hospitals for treatment. In addition to the problems regarding physical distance, travel to urban specialty healthcare services imposes financial burden on the patients and their families, since they have to pay for transportation, accommodation, and somehow make up for wages lost because of time taken off work (1, 3, 39). Besides, the transfer roads are often bumpy, which is uncomfortable for patients, and might influence, even exacerbate, certain medical conditions. Diagnosis and treatment are likely delayed for patients without the means to travel to specialty healthcare services in cities, resulting a further physical and mental adverse influence on both the patients and their family members (40, 41).

**Figure 3:**
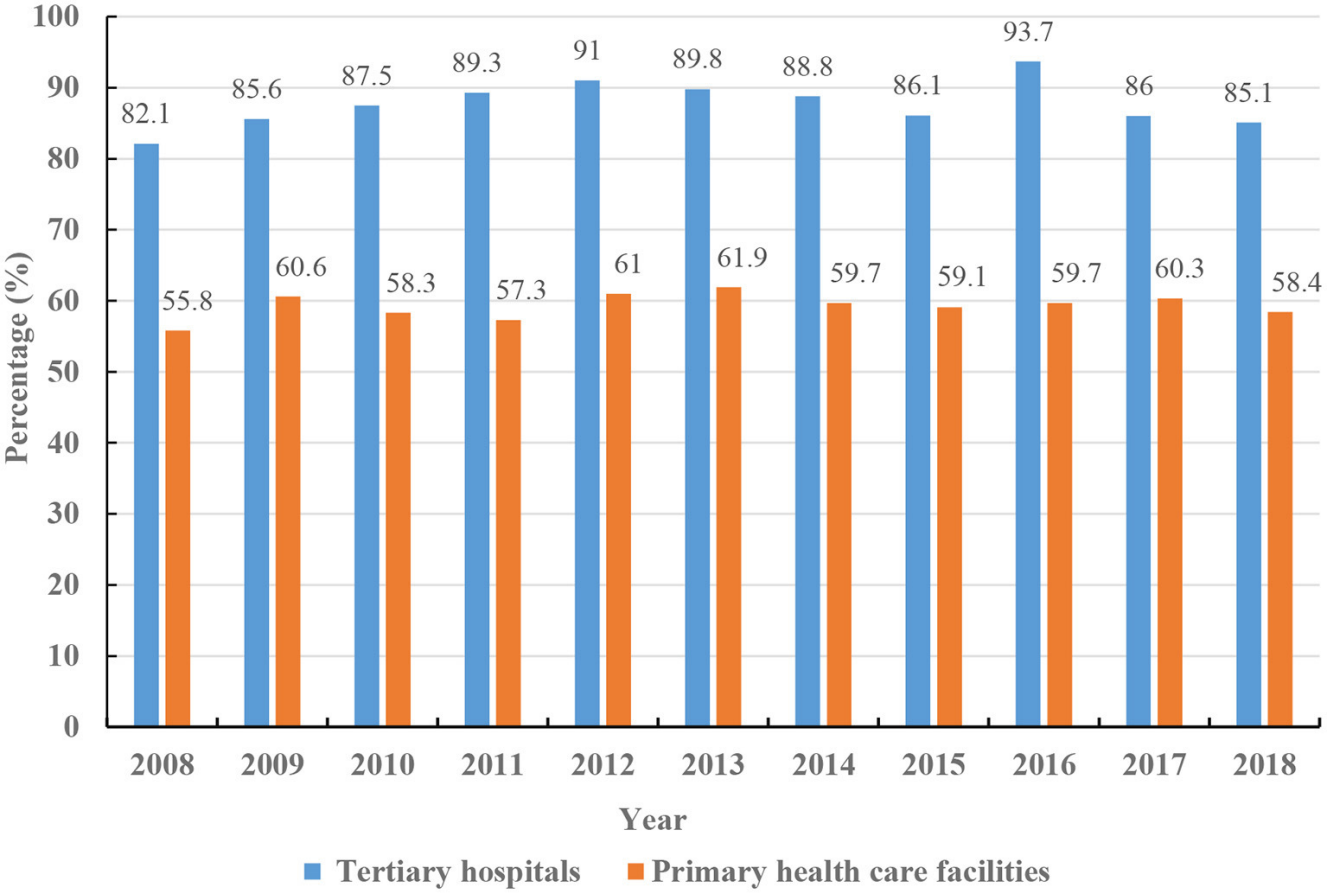
The bed use rate in urban tertiary hospitals and local primary healthcare facilities.

Patients in China are free to choose their healthcare facilities and doctors, then even for minor symptoms, lots of people tend to travel to municipal or provincial level hospitals for diagnosis and treatment (34). Although many diseases can be cured at primary hospitals at affordable prices with convenient access, many patients hesitate to use these facilities because they lack confidence in the local healthcare professionals and the quality of healthcare services provided there (34, 42). Indeed, skilled doctors in China tend to avoid working at remote rural facilities for both financial and professional reasons (34, 43). The preference for the top-tier urban hospitals has increased the bed tension and business burden, whereas the local county-level hospitals have plenty of available beds, which creates the contradictory co-existing phenomena of unequal geographical distribution and polarization between urban and rural healthcare resources, healthcare inefficiencies, and lack of access to specialty healthcare services (Figure 3) (33, 43). Telemedicine offers a feasible solution to the unequal allocation of medical resources, rural/urban gap in the capability and quality of disease diagnosis and treatment, and meets the public's urgent needs for high-quality healthcare services (1, 33, 34). In China, telemedicine is gradually becoming a key approach to respond to these problems.

### The History of Telemedicine

China initiated telemedicine in the late 1980s. In 1986, Guangzhou Ocean Shipping Company's hospital provided emergency transoceanic consultations for its ships' crews *via* radiotelegraphy, which was believed to be the first telemedicine practice in China (1). In 1988, the People's Liberation Army (PLA) General Hospital in Beijing conducted a remote neurosurgery case discussion *via* network satellite with a German hospital (3). Shanghai Medical University launched a pilot telemedicine project in 1995, and established one of the earliest telemedicine center in China (44). Since about that time, people began to search for medical help through the Internet. In the same year, there was a well-known instance that has become a classic telemedicine case at the international level. A Peking University student became ill for unknown reasons. Her classmates wrote her symptoms in English and emailed relevant foreign organizations for help through the Internet. They received more than 1,000 replies from 18 countries and regions around the world, which ultimately helped Chinese doctors to determine that the patient was afflicted with thallium poisoning (3).

In the late 1990s, from the theoretical exploration to gradual application, telemedicine in China experienced a rapid development period, and the representative milestones, namely, initiated telemedicine firstly or with the largest annual number of telemedicine services at national or provincial level, were summarized in Table 1. In 1997, the NHCC established the Chinese Jin-Wei Telemedicine Network, which provided teleconsultation and remote education services through satellite communication for hospitals in 21 provinces (3). The PLA and the National Health and Family Planning Commission of China (NHFPC) set up the Jun Wei II project (telemedicine network) in 2001. Since then, with the recognition and support of the central and local government, there were lots of telemedicine programmes initiated in other provinces, including Henan, Guizhou, Guangdong, and Fujian. Organizations such as the government, medical universities, hospitals, and even some private companies, sponsored their own telemedicine networks in succession, and made telemedicine a routine healthcare service (1, 3, 45). It has been reported that as of 2019, almost all of the 31 provinces and municipalities in mainland of China have established their own regional telemedicine centers (33, 46, 47).

**Table 1:** The development history of telemedicine in China.

**Year**	**Telemedicine milestone(s)**
1986	Guangzhou Ocean Shipping Company's hospital provided emergency transoceanic consultations for its ships' crews *via* radiotelegraphy
1988	The People's Liberation Army (PLA) General Hospital conducted a remote neurosurgery case discussion *via* network satellite with a German hospital
1994	Shanghai Medical University successfully carried out a teleconsultation between the Huashan hospital and the Shanghai Jiaotong University
1995	In Beijing, a student named Ling Zhu became ill for unknown reasons and searched for help *via* the Internet, which helped her to prove that she was afflicted with thallium poisoning; in Shanghai, the Shanghai Medical University launched a pilot telemedicine project and established a telemedicine center; in Shandong Province, a girl named Xiaoxia Yang suffered from an unidentified disease and asked for help through the Internet, then she was diagnosed as a phagocytic bacterial infection in the muscles
1996	The First Affiliated Hospital of Zhengzhou University initiated the first telemedicine center in Henan Province
1997	The International Medical Network Committee was established by China Medical Foundation to promote the development of medical information and telemedicine; the Chinese Jin-Wei Telemedicine Network was established to provide telemedicine services through satellite communication for hospitals in 21 provinces
1999	The Ministry of Health developed a set of rules to regulate and supervise telemedicine services
2001	The PLA and the National Health and Family Planning Commission of China (NHFPC) set up the Jun Wei II project (telemedicine network); the NHFPC launched the Shuang-wei telemedicine network to provide remote medical education services for doctors and nurses all over the country
2010	The NHFPC issued a guidance document about the development of teleconsultation services for tumor pathology and the construction of a network for quality control
2014	The NHFPC published the opinions on promoting telemedicine services in medical institutions
2015	The NHFPC carried out pilot work of telemedicine policy in five provinces: Ningxia, Yunnan, Inner Mongolia, Guizhou, and Tibet
2018	Office of the State Council published the opinions on promoting the development of Internet & medical health; the NHFPC issued the Standard of Telemedicine Service Management; the NHFPC approved the construction of the National Telemedicine Center.

### Scale and Coverage of Telemedicine Services

In 2001, the Jun Wei II telemedicine network covered approximately 300 hospitals across the whole country, which performed about 1,800 teleconsultation cases per year (48). By the end of 2003, the telemedicine network had carried out teleconsultation and remote education services for more than 1,000 patients and 50,000 medical staff, respectively (3). Between 2003 and 2010, lots of provinces in China launched their own regional telemedicine networks, including the Xinjiang, Gansu, Ningxia, Fujian, and Yunnan (Table 2). All these provincial telemedicine platforms were supported by local government and are still in operation nowadays. Since 2010, the government has invested about USD 12.25 million in deploying and operating local telemedicine systems in 22 central and western provinces. During this process, 12 western provinces and 12 subordinate hospitals of the National Health Commission were brought together to establish a top-tier telemedicine network that included 110 tertiary hospitals, three secondary hospitals, and 726 county-level hospitals (49).

**Table 2:** The time and organization that firstly initiated telemedicine network/center in the 31 provinces and municipalities in mainland of China.

**Year**	**Provinces/municipalities**	**Organization(s)**
1996	Hunan	Xiangya Hospital Central South University
1996	Henan	The First Affiliated Hospital of Zhengzhou University
1997	Beijing	The People's Liberation Army (PLA) General Hospital, Beijing Union Medical College Hospital
1998	Shandong	Shandong Provincial Hospital
2000	Fujian	PLA Fuzhou General Hospital
2001	Sichuan	West China Hospital of Sichuan University
2001	Jiangxi	Jiangxi Provincial People's Hospital
2002	Shanghai	Huashan hospital
2002	Tibet	PLA Tibet Military Region General Hospital
2005	Guizhou	Affiliated Hospital of Zunyi Medical University
2006	Yunnan	SUNPA Telemedicine
2007	Gansu	Gansu Provincial Hospital
2007	Ningxia	General Hospital of Ningxia Medical University, People's Hospital of Ningxia Hui Autonomous Region
2008	Xinjiang	The First Affiliated Hospital of Xinjiang Medical University
2008	Zhejiang	The Second Affiliated Hospital of Zhejiang University School of Medicine
2010	Guangdong	Nanfang Hospital of Southern Medical University
2011	Anhui	The First Affiliated Hospital of Anhui Medical University
2011	Chongqing	The Second Affiliated Hospital of Chongqing Medical University
2011	Hubei	Union Hospital affiliated to Tongji Medical College of Huazhong University of Science and Technology
2011	Qinghai	Qinghai University Affiliated Hospital
2012	Guangxi	The First Affiliated Hospital of Guangxi Medical University
2012	Jilin	Jilin Province People's Hospital
2012	Inner Mongolia	Inner Mongolia People's Hospital
2012	Shaanxi	Shaanxi Provincial People's Hospital
2013	Hainan	Hainan General Hospital
2013	Liaoning	Dalian Municipal Central Hospital
2014	Hebei	Hebei General Hospital
2014	Jiangshu	Nanjing Drum Tower Hospital
2014	Tianjin	Tianjin Medical University, Tianjin University of Traditional Chinese Medicine
2016	Heilongjiang	Qiqihar Medical University
2018	Shanxi	The Second Hospital of Shanxi Medical University

According to the NHFPC, in 2013, 2,057 healthcare facilities sponsored telemedicine services throughout China, and regional top-tier hospitals of many provinces established separate telemedicine platforms themselves (49). In 2017, 22 provinces had provincial telemedicine platforms comprised of about 13,000 healthcare facilities in more than 1,800 counties, which conducted about 60 million person-time telemedicine services (33). As of 2018, China's telemedicine network had covered more than 3,000 hospitals across the country, with more than 60 specialty areas, and there were six provincial telemedicine centers have completed business interconnection with each other (50). The appointment period of teleconsultation was shortened from 7 days to 2 days, and for the emergency and critical cases the time was not more than 2–4 h. For emergency rescue, a brand new remote collaborative information channel can be established within 30 min. It has been suggested that, in 2019, the market size of telemedicine in China reached about USD 2.68 billion (51).

Cui et al. investigated the general implementation and application of telemedicine in 29 provinces, autonomous regions, and municipalities across China. The authors reported that in 2017, among the investigated 185 tertiary hospitals, 253 secondary hospitals, and 26 primary hospitals, there were 161 (87.03%), 187 (73.91%), and nine (34.62%) hospitals have conducted telemedicine services, respectively (47). As of June 2020, the number of Internet medical users in China had reached 276 million, accounting for 29.4% of the total Internet users, and 26.4% of Internet users had purchased medicines and health equipment online (52, 53). For example, in 2020, the revenue of the teleconsultation business carried out by Alibaba Health reached 5.93 million dollars, implying a year-on-year increase of 221.2%. During the COVID-19 epidemic, 17.9% of Internet users have used telemedicine services such as remote registration and teleconsultation. Affected by the epidemic, the remote health consultation, diagnosis and treatment services of telemedicine platforms in China increased by more than 20 times, and the number of remote prescriptions increased by nearly 10 times (52).

### Procedure of Telemedicine Service

**Figure 4:**
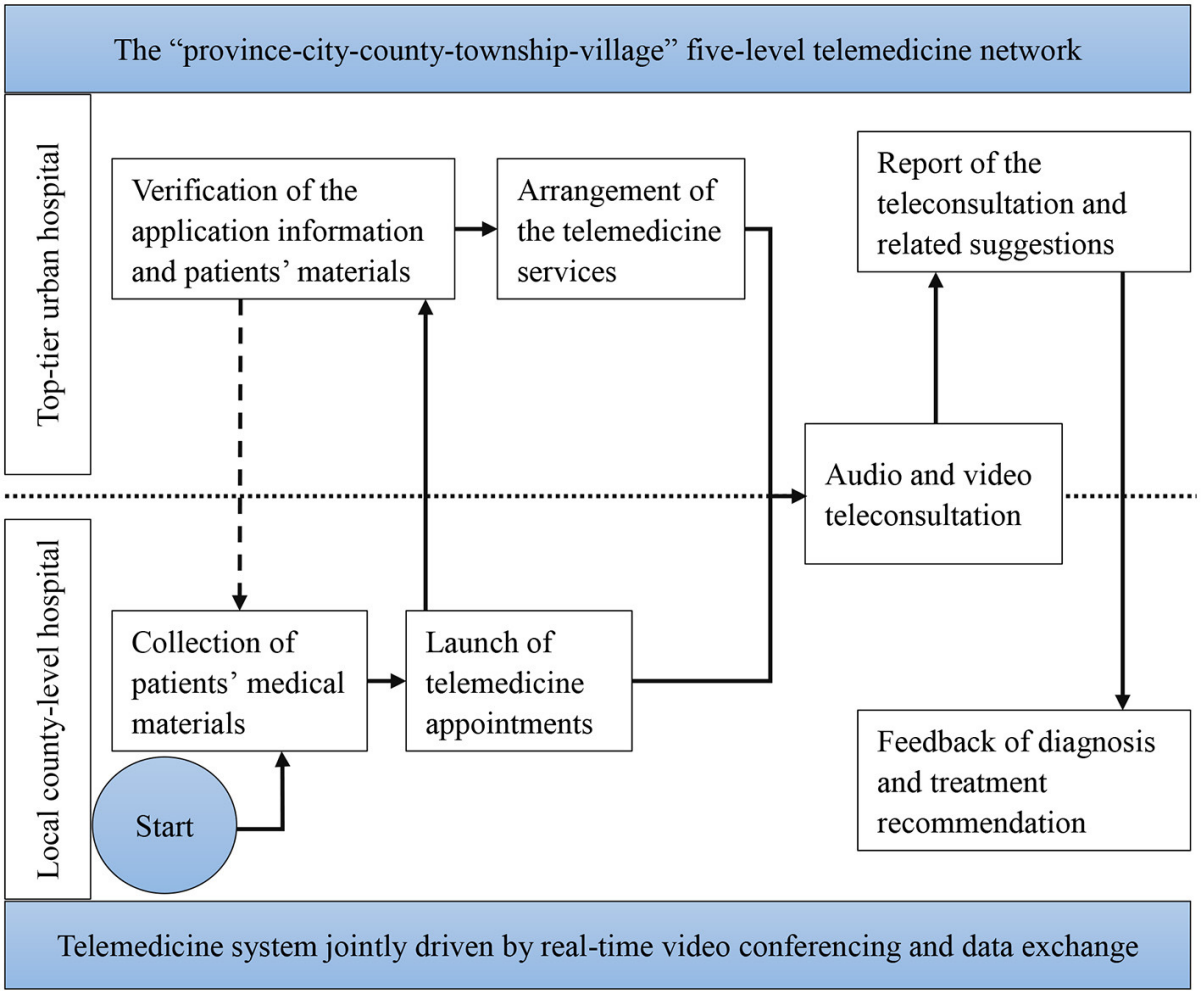
The general operation procedure of telemedicine services in China.

Telemedicine is an increasingly popular way to reduce the unequal distribution of healthcare resources in China. Generally, there are three common telemedicine service modes worldwide: medical institution-to-medical institution (B-B), medical institution-to-patient (B-C), and medical institution-to-telemedicine enterprise-to-patient (B-B-C) (4, 47). In China, the telemedicine service model is mainly B-B (47). For B-B model in China, although the operation procedures of telemedicine services in different telemedicine platforms may discrepant, the general process of a telemedicine service is similar as following (Figure 4). Through local sub-center (county-level hospital) of the telemedicine network, patients (applicants) can apply for top-tier urban hospital (provider) healthcare services, and the local medical institution uploads the patients' electronic medical records (EMR) and set teleconsultation appointments. After receiving the materials, the top-tier hospital being consulted verifies the information, arranges for experts and teleconsultation activities, and prepares the site and facilities for telemedicine services. Then, through the telemedicine system jointly driven by real-time videoconferencing and data exchange, physicians of the patients side report the medical histories and transfer relevant materials to the experts.

Based on the information from the EMR data, picture archiving and communication system (PACS), laboratory information system (LIS), electrocardiograms (ECG) data, and the physicians' statements, the experts diagnose the patients and provide treatment advice (5). Multi-center teleconsultation and case discussion are performed for complex cases or intractable diseases. Last, diagnostic conclusions and treatment plans are recommended, and necessary feedback is provided to the local physicians. When the process is complete, all relevant information and records are organized and filed. The superior hospital continuously communicates with the local doctors using follow-up system of the telemedicine platform to regularly assess patients and update treatment plans in time. During the telemedicine services, for the business processes of relevant subjects from different levels, such as local healthcare facilities, referral system, and the top-tier hospitals were summarized in Table 3.

**Table 3:** The business processes of relevant subjects from different levels during the telemedicine services.

**Subject**	**Role**	**Business process**
Local healthcare facilities	Applicants	According to the patient's actual condition and personal requirements, apply for teleconsultation to the top-tier hospitals through telemedicine network After the invitee accepts the application, the doctor in charge of the patient in the local healthcare facilities uploads the patient's EMR to the top-tier hospitals The doctor discussed the patient's condition with the consultation experts, and formulates and optimizes the patient's treatment plan After telemedicine services, make a record of the teleconsultation, and provide feedback on the follow-up patient treatment effect and outcome to the top-tier hospitals
Referral system	Coordinator /administrator	It is a functional module of the telemedicine platform For the local healthcare facilities, it receives teleconsultation applications, sets and notifies teleconsultation appointments, and coordinates the referral of serious or intractable cases to the top-tier hospitals For the top-tier hospitals, it invites teleconsultation experts, coordinates teleconsultation appointments, and arranges the reception of patients referred from the local healthcare facilities
The top-tier hospitals	Providers	Through the telemedicine network, accept teleconsultation applications from the local healthcare facilities, organize specific experts and arrange venues for the teleconsultations Guiding the doctor in charge of the patient in local healthcare facilities to develop and improve the patient's treatment plan Making necessary records for the teleconsultation, and following-up the treatment effect and outcome of the patient Evaluating the quality and effectiveness of the teleconsultation, and based on which to further improve the capabilities of telemedicine service.

### Henan Province Telemedicine Center (HTCC)

HTCC began developing its platform and practical operations in 1996, and was set as the National Telemedicine Center in January 2018. Currently, it has firstly developed and applied the “nation-province-city-county-township-village” six-level telemedicine network in China, covering the entire Henan and connecting with several other provinces across the country (5). As an open and sharing comprehensive platform, HTCC provides various telemedicine services to 35 tertiary hospitals, nearly 300 secondary hospitals in Henan Province, and, with more than 1,000 teaching and coordination networks and teleconsultation sub-centers throughout the country, which enables the smooth dissemination of healthcare resources to underserved areas (5, 39).

To date, the HTCC has annually performed almost 30,000 teleconsultations, more than 1,000,000 remote specialty diagnoses, trained about 300,000 medical staff in approximately 300 remote education venues, and achieved remarkable implementation effects and socioeconomic benefits (5, 39, 54). For example, in 2017, in terms of hospitalization costs and food- and travel-related expenses, compared with transferring to top-tier hospitals for treatment, patients remained at primary hospitals for treatment with the help of telemedicine can save them approximately USD $17.5 million (5).

## Discussion

Based on telemedicine networks, the connections and information exchanges between top-tier hospitals and their subordinate facilities enable high-quality healthcare resources to be transmitted across space and time. Thus, telemedicine has become increasingly popular in recent years, especially for developing countries and regions. In the present study, the necessity, development history, scale and coverage, operation procedure of telemedicine in China were investigated, from both national and regional levels. According to our knowledge, this is the first time that the comprehensive account of telemedicine in China was explored.

Telemedicine enables people in underserved areas to overcome geographical and temporal limitations and obtain specialty healthcare services similar to those they would receive in cities. The main pattern of telemedicine service in China is B-B model, based on telemedicine networks, lower-level healthcare facilities are connected with those upper-level ones, and patients make the appointment of telemedicine services through the doctors of the primary medical institutions. Patients just need to visit to the local community health service centers or county-level hospitals to apply for and receive teleconsultations and specialty diagnoses from skilled experts at tertiary hospitals (5, 47). Teleconsultation specialists spend 10 or more minutes discussing the materials from patient's EMR, PACS, LIS, and ECG with the local physician, then, provide diagnosis and treatment advice. Oppositely, most of the in-person clinic consultations of patients at top-tier hospitals last only a few minutes, which might be too short to fully understand and accurately diagnose a patient. This characteristic of telemedicine may increase the satisfaction of patients with healthcare services (34, 39, 47).

Telemedicine is often a less expensive, more convenient, and timesaving way to obtain healthcare without indirect costs than in-person hospital visits (1, 3, 55). Through telemedicine network, local healthcare facilities can directly diagnose and treat common diseases with the assistance of the professionals from tertiary hospitals. Thus, the top-tier hospitals have more time and attention to focus on the serious and intractable cases, which can help improve the appropriate dissemination of high-quality healthcare resources (33, 43, 56). For instance, telemedicine has been playing a particularly salient role in the COVID-19 epidemic in China (18). The COVID-19 limited the ability of healthcare specialists to work on site, while telemedicine was not restricted by this situation (57, 58). Besides, during the telemedicine services such as teleconsultation, specialty diagnoses, surgical techniques teaching, and occupational education, primary hospitals' staff might learn advanced experience and techniques used at top-tier hospitals. This may improve the quality and capacity of their services, shrink the gap between urban and rural healthcare, increase the use rate of local hospitals' beds, and rationalize the distribution of patients throughout the telemedicine network, which could ultimately help strengthen China's hierarchical medical system (18, 39, 46, 54).

### Challenges Faced by Telemedicine

In recent years, although telemedicine in China has developed rapidly in terms of system development, network connection, and scale expansion, it is still in the exploratory stage, and many problems remain. First, the telemedicine systems built in different regions vary in their technical framework, data transmission protocols, and application programming interfaces, which makes it challenging to effectively integrate multi-source heterogeneous data across regions and healthcare facilities (5, 59). This problem might create “data islands” that limit information sharing and cross-regional telemedicine services (4, 8, 33). Second, telemedicine service procedures and models vary across medical institutions, non-uniform standards may restrict the regional, national or international communication and popularization of telemedicine (8, 60). Third, it is difficult to clarify and unify the costs of various telemedicine services across the country, and most of these services are not covered under health insurance, this may prevent people from choosing telemedicine services (8, 34, 61). Fourth, in some provinces, the existing telemedicine networks and services heavily rely on government or project funding, the return on investment is relatively long term, and the mechanisms of long-term sustainable operation of telemedicine are not clear (2, 3, 34).

It has been discussed that there are four types of digital divide barriers, mental, material, skill, and usage, that may limit the further applications of telemedicine (62, 63). The authors suggested, to date, many people are still reluctant to choose telemedicine services because of at least six factors, including lack of trust, lack of support, lack of accessibility, user-unfriendly platforms, digital illiteracy, and inequalities related to gender, age and social groups (62). However, this situation may be somewhat different in China. Due to the great progress of education popularization in recent years, in 2020, there were only 37.75 million illiterate people in China, with an illiteracy rate 2.67% (37). According to the report on China's Internet development, as of December 2021, the number of Internet users in China was 1.032 billion, and the Internet penetration rate reached 73.0% (64). Among the Internet users, the ratio of males to females was 51.5:48.5, which is basically the same as the ratio in the overall population (64). For telemedicine in China, the software and hardware facilities involved in telemedicine are usually purchased and configured uniformly by the regional top-tier public hospitals relying on the support of national or local governmental projects, meaning that the infrastructure required for telemedicine is well guaranteed. Besides, since the B-B model of telemedicine services is adopted mostly in China, during a telemedicine service, patient is with the help of local doctors to apply for telemedicine services to top-tier hospitals through primary healthcare facilities (5). Thus, strictly speaking, the service process is an expert-to-expert approach, and there are fewer restrictions on patients. In this context, it has been reported that the majority of both medical practitioners (68.4%) and patients (60.0%) hold positive attitudes toward telemedicine services (65). Accordingly, in terms of the coverage and scale of telemedicine services in recent years, the abovementioned four types of digital divide barriers may play a limited role in the further development and implementation of telemedicine in China.

Due to the relatively time-consuming and sophisticated process of telemedicine services, many clinicians are reluctant to spend time to understand and master telemedicine related technologies and operational procedures, which limits the application of telemedicine in more medical institutions. Thus, realistically feasible and clinically appropriate telemedicine systems and related applications should be developed and deployed. To date, in different hospitals or even in one medical institution for different diseases, the length of waiting time for telemedicine service appointments varies greatly. How to standardize the service process and promote the efficiency of telemedicine appointments has become a key to further improve the quality of telemedicine services (9, 66). Besides, current telemedicine services have not developed applicable ways to protect patients' privacy or ensure healthcare data security, and the rights of relevant stakeholders and allocation of responsibilities in the event of a medical dispute or malpractice are also immature (5, 34, 67). Lastly, during telemedicine services, the scope of the business, the appropriate disease types, supervision, assessment, and quality control need to be further considered. These concerns reveal the future directions for further development and implementation of telemedicine in China.

### Limitations

Several limitations of this study must be acknowledged. For each of the 31 provinces and municipalities in mainland of China, there are more than one healthcare facilities that have developed and implemented telemedicine services. However, in the present study, only the provincial organizations that firstly initiated telemedicine network or center were investigated. The telemedicine systems built in different regions and even in the same province are usually discrepant. Thus, only the general situations, such as the content, operation procedure, and scale of telemedicine services, were discussed. Further studies are needed to address these issues.

### Future Work

Because of ICTs improvement and the increasing public need for lower medical costs, telemedicine has important practical significance for China where high-quality healthcare resources are concentrated in megacities and remote rural areas have little, if any, healthcare resources. In terms of both the challenges and necessity of telemedicine in China, the future development and implementation directions of telemedicine include but not limited to the followings. Firstly, the price standards for specific telemedicine services should be introduced or improved, at least at regional level, and the government should include telemedicine in the scope of medical insurance. Secondly, the operation procedure of telemedicine should be optimized to adapt to the doctors' time, for example, the teleconsultation site not being restricted by fixed places, and physicians can perform telemedicine services at any place where they are convenient (9, 68). Thirdly, medical institutions can encourage doctors to use telemedicine through establishing relevant incentive mechanism, such as performance appraisal, social prestige, or financial bonuses. Fourthly, AI could be integrated into the existing telemedicine system to assist the early screening of diseases and diagnosis of patients from remote areas as well as realize intelligent triage when the applications of telemedicine service are submitted by primary hospitals (17, 66). This can reduce the workload of coordinators and doctors in charge of telemedicine services. Last but not the least, in addition to single teleconsultation, ideally, telemedicine should be employed as an intermediate bridge to achieve bidirectional referral, and connect local healthcare facilities with top-tier hospitals more closely. Therefore, with the assistance of telemedicine, common and mild diseases should be diagnosed, treated, and rehabilitated directly at local primary hospitals, while intractable cases can be referred quickly to top-tier tertiary hospitals for treatment.

### Implications

In terms of the mentioned challenges and future directions of telemedicine in China, progressive leadership at the national, provincial, and local levels should develop or improve policies that coalesce standards and regulations into coherent visions for telemedicine. At the national level, the departments of healthcare management can set comprehensive regulatory and legal frameworks for telemedicine services that across provincial borders. Provincial or local governments could supplement such policies to accord with local medical, socioeconomic, legal, and cultural needs. These measures will shift telemedicine in China from its current fragmented systems, various service procedures and models, limited or even null reimbursement mechanisms, and poor information security of development and implementation to one that benefits from uniform standards, procedures, and management within which stakeholders such as governments, funders, healthcare facilities, and patients can enjoy more welfare from telemedicine services. Besides, such environments with uniform regulations will also help researchers of different provinces in China better perform scientific studies, and then the improved evidence base can in turn refine policies. Thus, in China, the changes or updates of top-down telemedicine related policies and guidelines in the near future are necessary to further develop, implement, and evaluate need-based telemedicine services.

Similar to the situations in China, the major issues in the development and implementation of telemedicine may be also faced by other countries around the world. Specifically, aforementioned problems such as the “data island” between different telemedicine system due to disparate technology architectures and information transmission standards, data intercommunication and integration of heterogeneous medical information, cross region intercommunication of telemedicine services with various procedures and models, weak reimbursement mechanisms, and poor privacy and information security of patients, are all the global common issues that need to be addressed to further promote the development and popularization of telemedicine. Thus, the challenges, future directions, potential response policies, and suggestions discussed in the present study may provide significant references and inspirations for other regions or countries worldwide.

## Conclusions

Telemedicine is one positive response to China's Internet & Medical innovation strategy by reducing the unequal distribution of medical resources, improving healthcare in remote and impoverished areas, and meeting public's demands for superior healthcare services in different regions, especially for the remote underserved areas. Telemedicine is also an encouraging initiative that promotes the implementation of Healthy China strategy, and it is recognized as an effective way to ensure that everyone enjoys the equal rights to and opportunities for high quality healthcare services. Through improving the capacity, quality, and efficiency of healthcare in underserved areas and reducing the unequal distribution of medical resources, telemedicine can support the development of China's hierarchical medical system that offers initial diagnosis at the community, bidirectional referrals, acute and chronic treatment classifications, and upper and lower medical institution linkages, which plays an active role, at the regional or even national level, in solving the problems of the difficulty and high cost to access to medical services.

## Data Availability Statement

Publicly available datasets were analyzed in this study. This data can be found at: National Bureau of Statistics. The 2019/2020/2021 China Statistical Yearbook [In Chinese]. China statistics press. Internet: www.stats.gov.cn/tjsj/ndsj/ (accessed April 2, 2022).

## Author Contributions

JG conceptualized, designed, and initiated the study. JG, CF, and BC drafted the initial manuscript. ZF, LL, LW, QM, XH, and YZ involved in the development of methodology and discussion of article structure. JG and JZ reviewed and revised the manuscript. All authors read and approved the final manuscript as submitted.

## Funding

This work was supported by Natural Science Foundation of Henan Province of China (202300410409), Joint construction project of the Henan Province medical science and technology research plan (Grant Nos. 2018020120 and LHGJ20200331), and special funds of major science and technology project in Henan province (Grant No. 201400210400). The funders played no role in the design, development, or interpretation of the present work. The views expressed in the article are those of the authors and do not necessarily reflect the position of the funding bodies.

## Conflict of Interest

The authors declare that the research was conducted in the absence of any commercial or financial relationships that could be construed as a potential conflict of interest.

## Publisher's Note

All claims expressed in this article are solely those of the authors and do not necessarily represent those of their affiliated organizations, or those of the publisher, the editors and the reviewers. Any product that may be evaluated in this article, or claim that may be made by its manufacturer, is not guaranteed or endorsed by the publisher.

## Abbreviations

COVID-19: novel coronavirus 2019
ECG: electrocardiograms data
EMR: electronic medical records
HPHC, National Bureau of Statistics: Henan Province Health Commission
HTCC: Henan Province Telemedicine Center of China
LIS: laboratory information system
NHCC: National Health Commission of China
NHFPC: National Health and Family Planning Commission of China
PACS: picture archiving and communication system
PLA: People's Liberation Army
WHO: World Health Organization.
